# KIAA0101 and UbcH10 interact to regulate non-small cell lung cancer cell proliferation by disrupting the function of the spindle assembly checkpoint

**DOI:** 10.1186/s12885-020-07463-3

**Published:** 2020-10-02

**Authors:** Han Lei, Kun Wang, Tongying Jiang, Jingjing Lu, Xue Dong, Feilong Wang, Qiang Li, Liming Zhao

**Affiliations:** 1 Department of Pulmonary and Critical Care Medicine, Shanghai East Hospital, Tongji University School of Medicine, No. 150 Jimo Road, Pudong, Shanghai, 200120 P.R. China; 2 Department of Pulmonary and Critical Care Medicine, Shanghai General Hospital, Shanghai Jiao Tong University, Shanghai, 200080 China; 3 Department of Pulmonary and Critical Care Medicine, Shanghai East Hospital, Tongji University School of Medicine, No. 1800 Yuntai Road, Pudong, Shanghai, 200120 P.R. China

**Keywords:** Non-small cell lung cancer, Spindle assembly checkpoint, UbcH10, KIAA0101

## Abstract

**Background:**

Chromosome mis-segregation caused by spindle assembly checkpoint (SAC) dysfunction during mitosis is an important pathogenic factor in cancer, and modulating SAC function has emerged as a potential novel therapy for non-small cell lung cancer (NSCLC). UbcH10 is considered to be associated with SAC function and the pathological types and clinical grades of NSCLC. KIAA0101, which contains a highly conserved proliferating cell nuclear antigen (PCNA)-binding motif that is involved in DNA repair in cancer cells, plays an important role in the regulation of SAC function in NSCLC cells, and bioinformatics predictions showed that this regulatory role is related to UbcH10. We hypothesized KIAA0101 and UbcH10 interact to mediate SAC dysfunction and neoplastic transformation during the development of USCLC.

**Methods:**

NSCLC cell lines were used to investigate the spatial-temporal correlation between UbcH10 and KIAA0101 expression and the downstream effects of modulating their expression were evaluated. Further immunoprecipitation assays were used to investigate the possible mechanism underlying the correlation between UbcH10 and KIAA0101. Eventually, the effect of modulating UbcH10 and KIAA010 on tumor growth and its possible mechanisms were explored through in vivo tumor-bearing models.

**Results:**

In this study, we demonstrated that both UbcH10 and KIAA0101 were upregulated in NSCLC tissues and cells and that their expression levels were correlated in a spatial and temporal manner. Importantly, UbcH10 and KIAA0101 coordinated to mediate the premature degradation of various SAC components to cause further SAC dysfunction and neoplastic proliferation. Moreover, tumor growth in vivo was significantly inhibited by silencing UbcH10 and KIAA0101 expression.

**Conclusions:**

KIAA0101 and UbcH10 interact to cause SAC dysfunction, chromosomal instability and malignant proliferation in NSCLC, suggesting that UbcH10 and KIAA0101 are potential therapeutic targets for the treatment of NSCLC by ameliorating SAC function.

## Background

Non-small cell lung cancer (NSCLC) is currently one of the most common malignancies and accounts for a large proportion of deaths globally [1]. However, despite considerable advancements in theranostics in the past few decades, the overall mortality has remained relatively high [2]. Therefore, preclinical research on NSCLC still has a long way to go, and there is an urgent need for new therapeutic targets in the development of novel treatment strategies.

Aneuploidy caused by chromosome mis-segregation during mitosis is an important pathogenic factor in cancer [3]. Accurate chromosome segregation depends on bipolar attachment of kinetochores to microtubules and alignment along the equatorial plate during metaphase. Improperly attached kinetochores activate a highly conserved mitotic surveillance system called the spindle assembly checkpoint (SAC), which inhibits the onset of chromosome segregation until the improper attachment is rectified; this system ensures that chromosomes in the parental cell are equally and accurately assigned to two daughter cells [4, 5]. SAC dysfunction leading to aneuploidy during tumorigenesis as a result of changes in SAC-associated protein levels has been observed in various human cancers [6, 7]. In NSCLC, impaired SAC function and aneuploidy can be found in over 50% of cases [8]. Therefore, it is of great significance to further explore the molecular mechanisms that regulate SAC function and develop related factors as potential therapeutic targets for NSCLC.

UbcH10 is highly expressed in tissues from many different types of cancer, including NSCLC, and is closely related to disease progression and prognosis [9–12]. In recent years, a series of studies have suggested that UbcH10 plays an important role in tumorigenesis by regulating SAC function [13, 14]. However, the mechanism of UbcH10 and its ability to regulate NSCLC pathogenesis need to be further elucidated. KIAA0101 is another appealing oncogene that was recently validated as an independent prognostic factor in NSCLC [15–17]. By utilizing a bioinformatics database, we found that the coding sequences (CDSs) of KIAA0101 and the SAC member BubR1 are both located on chromosome 15q14–21. KIAA0101 also shares a common promoter with the APC/C coactivator Cdc20, suggesting that the biological function of KIAA0101 overexpression in NSCLC may be related to the SAC.

Based on the correlations between UbCH10, KIAA0101 and SAC function, we hypothesized that during the development of NSCLC, KIAA0101 may interact with UbcH10 to mediate SAC dysfunction and neoplastic transformation. To the best of our knowledge, this report is the first on the interaction between UbcH10 and KIAA0101 and on their synergistic effect on neoplastic transformation through effects on SAC function. These data may provide theoretical support for the development of novel therapeutic strategies targeting UbcH10 and KIAA0101 for the treatment of NSCLC.

## Methods

### Cell culture

The human NSCLC cell lines A549 and SK-MES-1 were purchased from the Cell Bank of the Chinese Academy of Sciences (CBCAS, Shanghai, China) and maintained in RPMI-1640 medium (Thermo Fisher, CA, USA) supplemented with 10% fetal bovine serum (FBS, Thermo Fisher, CA, USA). All cells were passaged by 0.25% trypsin digestion (Thermo Fisher, CA, USA) and cultured in an atmosphere of 5% CO2 at 37 °C in a humidified incubator (MCO-175, SANYO, Japan).

### Collection and immunohistochemical staining of clinical NSCLC samples

Twenty sets of NSCLC and adjacent normal tissues were obtained from the Shanghai Pulmonary Hospital between May 2017 and April 2018 (Table 1). The patients gave written informed consent, and the study was approved by the Ethics Committee of Shanghai Pulmonary Hospital. None of the patients had received chemotherapy before sample collection. Each sample was divided into the two parts, one for western blotting of UbcH10, KIAA0101, BubR1, Mad2 and CyclinB proteins, and one for immunohistochemistry of UbcH10 and KIAA0101. For western blotting, separate samples which were used for extracting total protein were rinsed with saline and transferred to 2 ml microtubes that were labeled and subsequently stored in liquid nitrogen. For immunohistochemistry, separate samples were placed in tissue clips immersed in 10% neutral formaldehyde. Then, paraffin embedding and pathological sectioning were performed successively. The embedded sections were cut into 4 μm slices and placed on a glass slide before immunohistochemical staining to examine UbcH10 and KIAA0101. The following antibodies were used: anti-UbcH10 (1:400) and anti-KIAA0101 (1:450) and HRP-labeled goat anti-rabbit (1:1200) (Abcam, Cambridge, UK).

**Table 1:** Clinicopathological features of 20 patients with NSCLC

No.	Sex	Age (years)	Pathological	Clinicalstage
1	M	54	Squamous carcinoma	II
2	M	49	Adenocarcinoma	III
3	F	64	Adenocarcinoma	II
4	M	63	Squamous carcinoma	II
5	F	52	Adenocarcinoma	III
6	F	68	Squamous carcinoma	II
7	M	66	Squamous carcinoma	II
8	M	72	Squamous carcinoma	III
9	F	70	Adenocarcinoma	III
10	F	69	Adenocarcinoma	II
11	F	57	Squamous carcinoma	III
12	M	73	Adenocarcinoma	II
13	M	59	Adenocarcinoma	III
14	M	49	Adenocarcinoma	II
15	F	72	Adenocarcinoma	III
16	M	68	Squamous carcinoma	II
17	F	76	Squamous carcinoma	III
18	F	48	Squamous carcinoma	III
19	F	72	Adenocarcinoma	III
20	F	68	Adenocarcinoma	II

Twenty sets of NSCLC and adjacent normal tissues were obtained from the Shanghai Pulmonary Hospital between May 2017 and April 2018. None of the patients had received chemotherapy before sample collection. The clinical stage was determined according to the 8th edition of TNM (UICC and AJCC)

*NSCLC* Non-small cell lung cancer, *M* Male, *F* Female

### Cell cycle arrest and release

SK-MES-1 cells in the logarithmic growth phase were seeded in 6-well plates at 5 × 10^5^ cells/well with RPMI-1640 medium containing 10% FBS and cultured overnight under normal conditions. Then, nocodazole (Sigma, CA, USA) at a final concentration of 1 μg/ml was added to the medium, and the cells were cultured for 18 h. Next, cycloheximide (CHX, Sigma, CA, USA) at a final concentration of 10 μg/mL was added to the medium, and the cells were cultured for another 5 h; some cells were collected every hour. UbcH10 and KIAA0101 expression in the cells harvested at different time points was detected by western blotting.

### Immunofluorescence assay

SK-MES-1 cells in the logarithmic growth phase were fixed with 4% formaldehyde in phosphate-buffered saline (PBS), permeabilized with 0.4% Triton X-100 in PBS, incubated with blocking buffer (10% donkey serum in PBS), and stained overnight at 4 °C with primary antibody. Then, the cells were incubated for 1 h at room temperature with secondary antibodies. Hoechst 33342 (Thermo Fisher, CA, USA) was used to stain nuclear DNA. The following antibodies were used: anti-UbcH10 (1:200) and anti-KIAA0101 (1:350), the FITC or Alexa Fluor-labeled goat anti-rabbit were used as secondary antibodies (1:1200, Abcam, Cambridge, UK).

### Genetic intervention using a lentiviral approach

SK-MES-1 and A549 cells in the logarithmic growth phase were seeded into 6-well plates at a density of 5 × 10^5^ cells/well. On the following day, the cells were infected with virus encoding UbcH10(Lv-UbcH10) or silencing KIAA0101(Lv-shRNA-KIAA0101 contains a siRNA sequence 5′-GACCTGAGGTATAAGCTCT-3′) or control (Lv-control) at a multiplicity of infection (MOI) of 10. The infection efficiency was assessed by observing and analyzing green fluorescent protein (GFP) fluorescence 72 h after infection with an inverted fluorescence microscope (IX71-F22, Olympus, Japan). The infection rate was estimated by dividing the number of cells expressing GFP by the total number of cells in each view. Total RNA and protein were isolated from the cells and subjected to real-time quantitative PCR (RT-qPCR) and western blotting to determine UbcH10 and KIAA0101 mRNA and proteins.

### Cell cycle analysis

SK-MES-1 and A549 cells infected with recombinant lentiviruses (Lv-NC contains a mistranslated sequence 5′- GAAGCCAGATCCAGCTTCC-3′ or Lv-shRNA-UbcH10 or Lv-shRNA-KIAA0101 contains a siRNA sequence 5′-GGAGGACAAATACGCAATG-3′ or Lv-shRNA-UbcH10 combined Lv-shRNA-KIAA0101) for 72 h were trypsinized, washed twice with PBS, and fixed with 70% ethanol at 4 °C overnight. The fixed cells were washed twice with PBS, resuspended in 100 μl propidium iodide (50 μg/ml and 100 μg/ml ribonuclease A in PBS), and incubated at room temperature for 30 min. The cell suspensions were detected by a FACSCalibur flow cytometer (BD Biosciences, NJ, USA).

### Cellular proliferation assay

SK-MES-1 and A549 cells infected with recombinant lentiviruses for 72 h were trypsinized and seeded into 96-well plates at a density of 1 × 10^5^ cells per well. The cells were cultured under normal conditions, and cell viability was examined using a Cell Counting Kit-8 (CCK-8) assay at 24, 48, and 72 h. Briefly, 10 μl CCK-8 solution (Dojindo, Japan) was added, and the cells were cultured under normal conditions for an additional 4 h before the absorbance at 450 nm was measured.

### Coimmunoprecipitation analysis

The coding sequence of KIAA0101 was amplified from human cDNA and cloned into the pcDNA3.1-HA expression vector (Invitrogen) to construct the wild-type (wt) KEN box vector pcDNA-HA-wt-KIAA0101 (wt KEN box: 5′-AGAAAGGTGCTT-3). Then, the mutant KEN box vector, pcDNA-HA-mt-KIAA0101 (mutant KEN box: 5′-GAAAAGGTTGCT-3′), was constructed through point mutation. SK-MES-1 cells transfected for 48 h with pcDNA-HA-wt-KIAA0101 or pcDNA-HA-mt-KIAA0101 by using Lipofectamine 2000 (Thermo Fisher, CA, USA) were lysed at 4 °C in ice-cold immunoprecipitation assay lysis buffer (Pierce, USA) for 10 min, and the resulting cell lysates were centrifuged for 3 min at 12,000 g. A negative control (NC) group was established that contained lysates from normal cells that were incubated with IgG. Before coimmunoprecipitation, samples containing equal amounts of protein were precleared with protein A or G agarose/Sepharose beads (Abcam, CA, USA) at 4 °C for 3 h and subsequently incubated with irrelevant IgG or anti-HA antibody (Santa Cruz, 2 μg/ml) in the presence of protein A or G agarose/Sepharose beads (Abcam, CA, USA) for 2 h at room temperature or overnight at 4 °C with gentle shaking. After incubation, the agarose/Sepharose beads were extensively washed with PBS, and the proteins were eluted by boiling in 2 × SDS sample buffer before separation by SDS-PAGE. Subsequently, the target protein was identified by western blotting.

### Animal xenografts

Animal studies were performed in accordance with ARRIVE guidelines. Sixty female athymic nude mice (Shanghai Slake Laboratory Animal Co., Ltd.; age, 10 weeks; average weight, ~ 20 g) were housed at 23 °C in a humidified atmosphere and a 12-h-light/dark cycle, with standard rodent chow and water ad libitum at the Second Military Medical University Animal Experiment Center, where the implantation experiment was performed. All the protocols were approved by the University Experimental Animal Ethics Committee. SK-MES-1 cells (1 × 10^5^) were suspended in 200 μl medium and injected subcutaneously into the flanks of mice. Two weeks after inoculation, subcutaneous tumors were visible, and these tumors were approximately 2.5 mm in diameter 2 weeks after inoculation. All animals were randomly divided into 5 groups (12 mice per group): the model group, NC group, UbcH10-shRNA group, KIAA0101-shRNA group, and co-shRNA group. In the intervention groups, each animal received 50 μl recombinant lentivirus (5 × 10^7^ IFU) twice a week (on Monday and Thursday) starting at the second week and continuing for 4 weeks, while the model group received the same volume of saline. The diameter of each tumor was measured weekly starting at the second week, and the data were used to plot tumor growth curves. At the end of the gene intervention (4 weeks), mice were killed by cervical dislocation under deep anesthesia by diethyl ether, and the subcutaneous tumors were stripped and used to detect UbcH10, KIAA0101, BubR1, Mad2 and CyclinB expression detected by western blotting.

### RT-qPCR

Total RNA was isolated with TRIzol Reagent (Thermo Fisher, CA, USA) according to the manufacturer’s instructions and was reverse transcribed into cDNA using M-MLV Reverse Transcriptase and the Random9 primer (Takara, Dalian, China). The following specific primers were used for PCR: UbcH10-forward 5′-GGCTACCCTTACAATGCGCCC-3′ and UbcH10-reverse 5′-CCTGACATCATACAGGGC-3′; KIAA0101-forward 5′-AACATAGCGTAAACCCTATC-3′ and KIAA0101-reverse 5′-CCTTGTTAGGCAGGATGGTCTC-3′; and β-actin-forward 5′-CCTGTACGCCAACACAGTGC-3′ and β-actin-reverse 5′-ATACTCCTGCTTGCTGATCC-3′. RT-qPCR was performed using the SYBR Premix Ex Taq kit and ABI7500 System (Thermo Fisher Scientific, CA, USA). One microliter of cDNA was used as the template. The mRNA levels of target genes were normalized to those of the endogenous housekeeping gene β-actin using the 2^-ΔCt^ method.

### Western blotting

Total protein was extracted from the cells using the M-PER mammalian protein extraction reagent or from tissues using the T-PER tissue protein extraction reagent (Pierce, IL, USA). Equal amounts of protein (12 μg per lane) as estimated by a bicinchoninic acid protein assay kit (Pierce, IL, USA) were separated by 11% SDS-PAGE and then transferred onto nitrocellulose membranes. The blots were probed with rabbit monoclonal antibodies against UbcH10 (1:200), KIAA0101 (1:500), BubR1 (1:600), Mad2 (1:250), CDC25(1:400), CDK1 (1:300), CDK7 (1:500), CyclinB (1:350) and β-actin (1:1000) (Abcam, CA, USA), followed by incubation with secondary HRP-conjugated goat anti-rabbit antibody (Abcam, CA, USA). After a washing step, the bands were detected by chemiluminescence and imaged on X-ray films. β-Actin was used as an endogenous reference for normalization.

### Statistical analysis

All statistical analyses were performed using SPSS 20.0 (SPSS, Chicago, IL, USA). The data are expressed as the mean ± standard deviation (SD). Differences between groups and the control were analyzed by Tukey’s post-hoc test. *P* < 0.05 was considered to indicate a significant difference. All data were obtained from three independent experiments.

## Results

### UbcH10 and KIAA0101 expression coincided with the expression of SAC components and cell cycle-associated proteins in NSCLC tissues

**Fig. 1:**
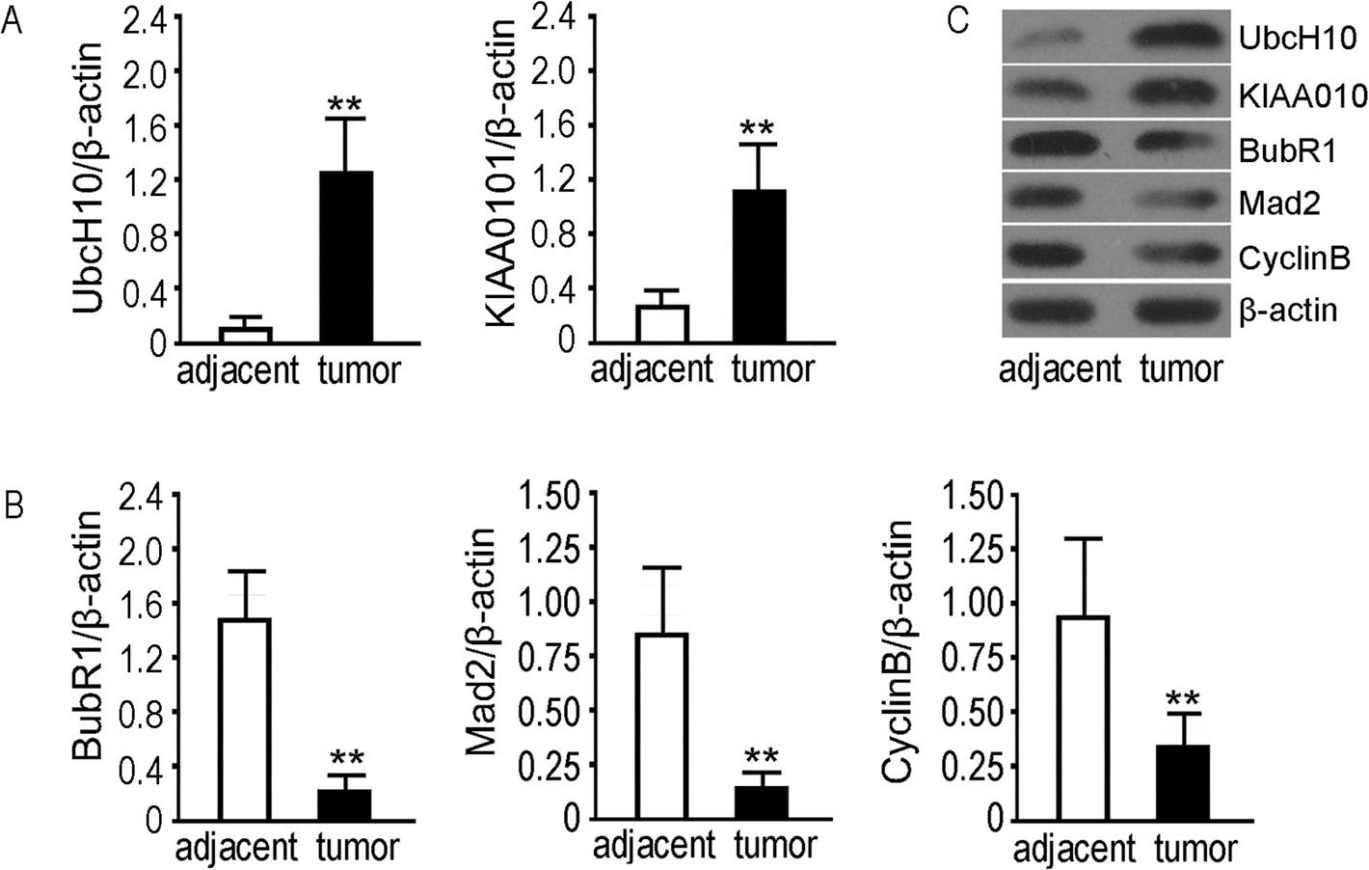
Western blotting for expression of UbcH10, KIAA0101 and the SAC components BubR1, Mad2 and CyclinB in NSCLC tumor and adjacent tissues. The sample size was 20 (*n* = 20). The information of enrolled patients is detailed in Table 1. The adjacent normal tissue was removed no less than 2.5 cm away from the edge of the tumor. The protein molecular weights are UbcH10 (21 kDa), KIAA0101 (18 kDa), BubR1(148 kDa), Mad2 (25 kDa) and Cyclin B (52 kDa); β-actin (43 kDa) was a reference protein. **a**, **b** Analysis of differential protein expressions between groups. The y-coordinate of bar chart represents the relative radio optical density values of proteins to β-actin. The data are expressed as the mean ± SD, **p<0.01 vs adjacent group. **c** Scanned target protein bands are shown

The results of protein detection showed that expressions of UbcH10 and KIAA0101 were significantly higher in NSCLC tumors than in adjacent normal tissues (*p*<0.01) (Fig. 1a), which is consistent with the findings of previous studies [12, 16]. Meanwhile, we examined the SAC components BubR1 and Mad2 and the cell cycle-associated protein CyclinB and found that all they were significantly lower in NSCLC tumors than in adjacent normal tissues (*p*<0.01) (Fig. 1b). The results indicated that UbcH10 and KIAA0101 were overexpressed in NSCLC tumors, which related to the expressions of SAC components and cell cycle-associated proteins.

### Spatial-temporal correlations between UbcH10 and KIAA0101

**Fig. 2:**
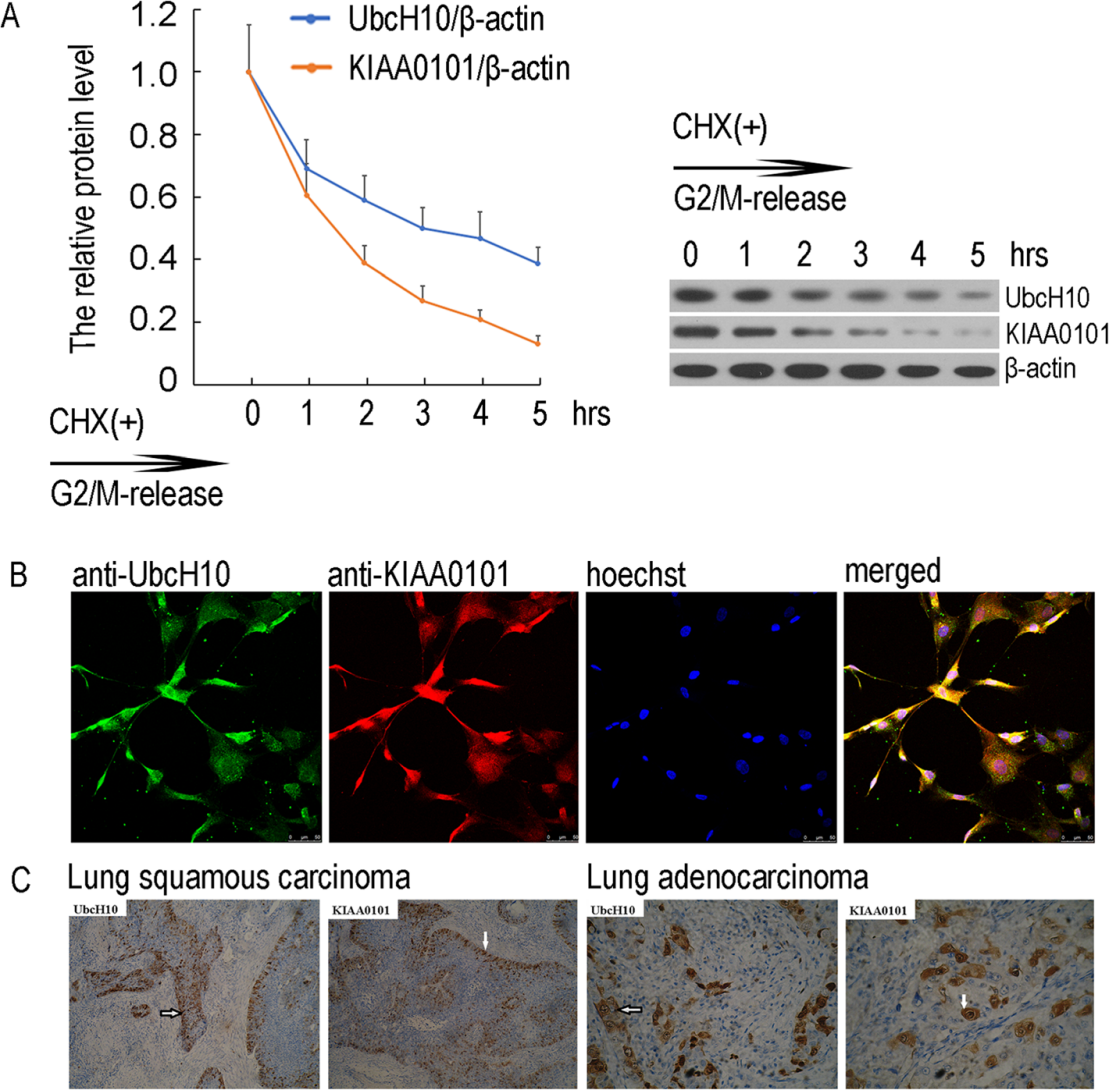
Correlation of UbcH10 and KIAA0101 expression in NSCLC cells and tissues. **a** The temporal correlation between UbcH10 and KIAA0101 in SK-MES-1cells as detected by western blotting. Cells were arrested in the G2 phase with Nocodazole (1 μg/ml) and then harvested at 0, 1, 2, 3, 4 and 5 h after release into the medium. Total proteins were extracted, and UbcH10 and KIAA0101 expression was detected by western blotting with β-actin serving as a reference, the relative expression of the target protein was expressed as the optical density ratio of the target protein to β-actin. Low, scanned target protein bands, top, the protein expression curve over time. **b** The spatial correlation between UbcH10 and KIAA0101 in SK-MES-1 cells as detected by immunofluorescence is shown as follows: cell nuclei are stained blue by Hoechst, and green and red labels show UbcH10 and KIAA0101 proteins, respectively. The magnification was 200×. **c** The spatial correlation between UbcH10 and KIAA0101 in NSCLC tissues as detected by immunohistochemistry is shown. Five fields were randomly selected for comparison

After nocodazole treatment of SK-MES-1 cells was terminated, UbcH10 and KIAA0101 protein contents gradually declined, suggesting a temporal correlation of the expression of these two proteins (Fig. 2a). We then observed the spatial correlation of KIAA0101 and UbcH10 in SK-MES-1 cells in G2/M phase through an immunofluorescence assay and found that these two proteins were colocalized (Fig. 2b). To further validate this finding, we performed immunohistochemical staining on tissue samples obtained from patients with lung squamous cell carcinoma or lung adenocarcinoma and observed the spatial colocalization of UbcH10 and KIAA0101 in SK-MES-1 cells and both lung squamous cell carcinoma and lung adenocarcinoma samples (Fig. 2c). Identical experimental data were obtained in A549 cells (data not shown). The results indicated that UbcH10 and KIAA0101 expression is spatially and temporally correlated in NSCLC cells and tissues.

### UbcH10 and KIAA0101 expression correlated at the transcriptional level

**Fig. 3:**
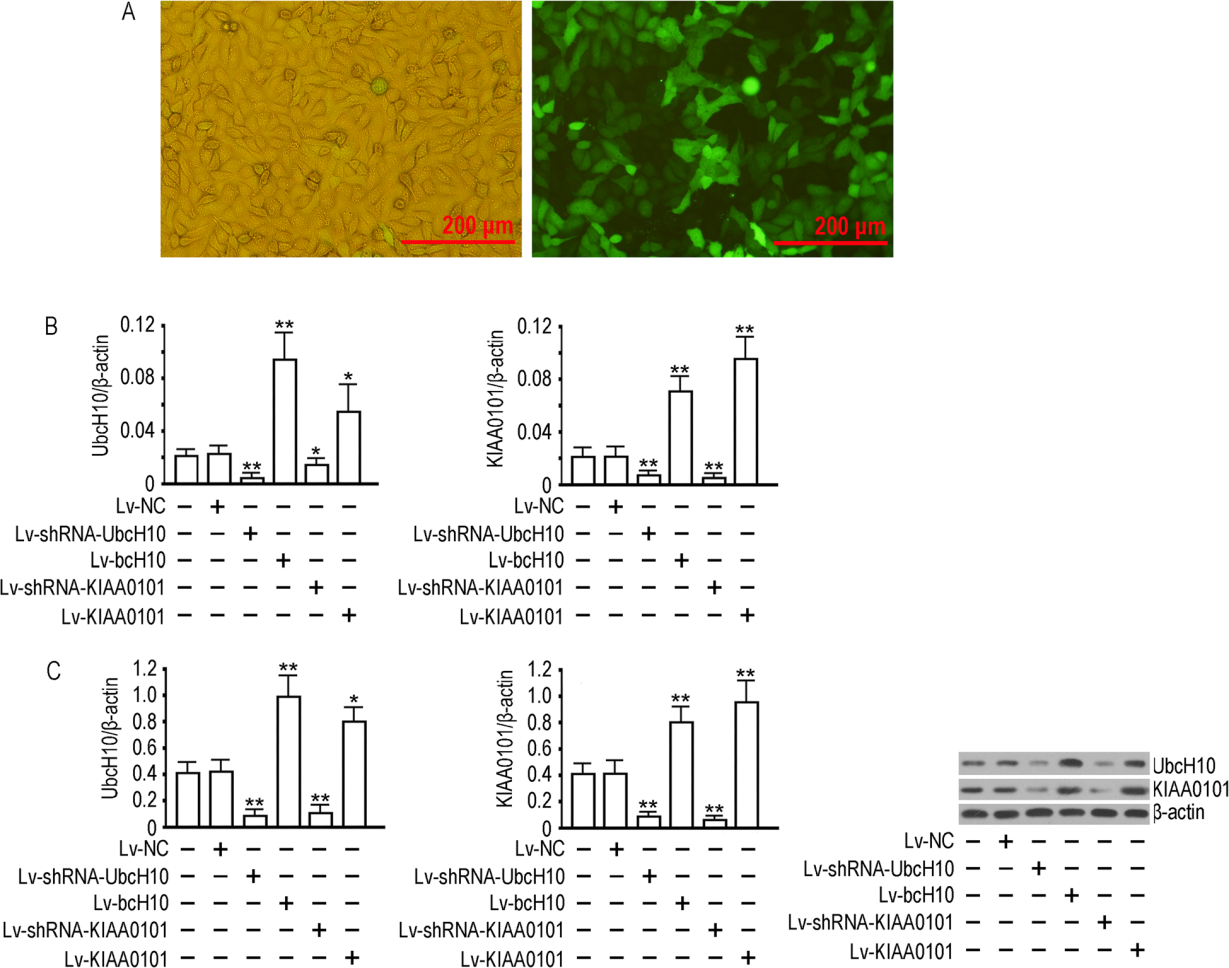
Lentivirus-mediated gene intervention in SK-MES-1 cells: **a** Detection of the lentiviral infection efficiency of SK-MES-1 cells: 72 h after infection with the recombinant virus (MOI = 10); the effectiveness was estimated by comparing the number of cells with fluorescent marker GFP expression (right) with the number of cells in the field of visible light (left) in the same field of view; **b** the relative content of UbcH10 and KIAA0101 mRNA in each group was detected 72 h after lentivirus infection with d β-actin used as the reference gene, and the difference between groups was calculated by the 2^-ΔΔCt^ method; and **c** UbcH10 and KIAA0101 expression was detected 72 h after virus infection. Three biological replicates of all experiments were performed, and the data are expressed as the mean ± SD, **p < 0.01 and *p < 0.05 vs cell group

To further elucidate the correlation between UbcH10 and KIAA0101, we regulated UbcH10 and KIAA0101 in SK-MES-1 cells using lentiviral transfection. Our lentiviral assay system achieved highly efficient transfection of tumor cells (Fig. 3a). RT-qPCR results showed that overexpression of either UbcH10 or KIAA0101 led to increased mRNA levels of both UbcH10 and KIAA0101 (*p*<0.01 compared to the control group), and inhibiting the expression of either UbcH10 or KIAA0101 significantly suppressed the mRNA levels of both UbcH10 and KIAA0101 (*p*<0.01 compared to the control or NC group) (Fig. 3b). Similar results were obtained by western blotting (Fig. 3c). Identical experimental data were obtained in A549 cells (data not shown). These results indicated that UbcH10 and KIAA0101 expression levels are correlated at the transcriptional level in NSCLC cells.

### UbcH10 and KIAA0101 coordinated to regulate SAC function, the cell cycle and the proliferation of NSCLC cells

**Fig. 4:**
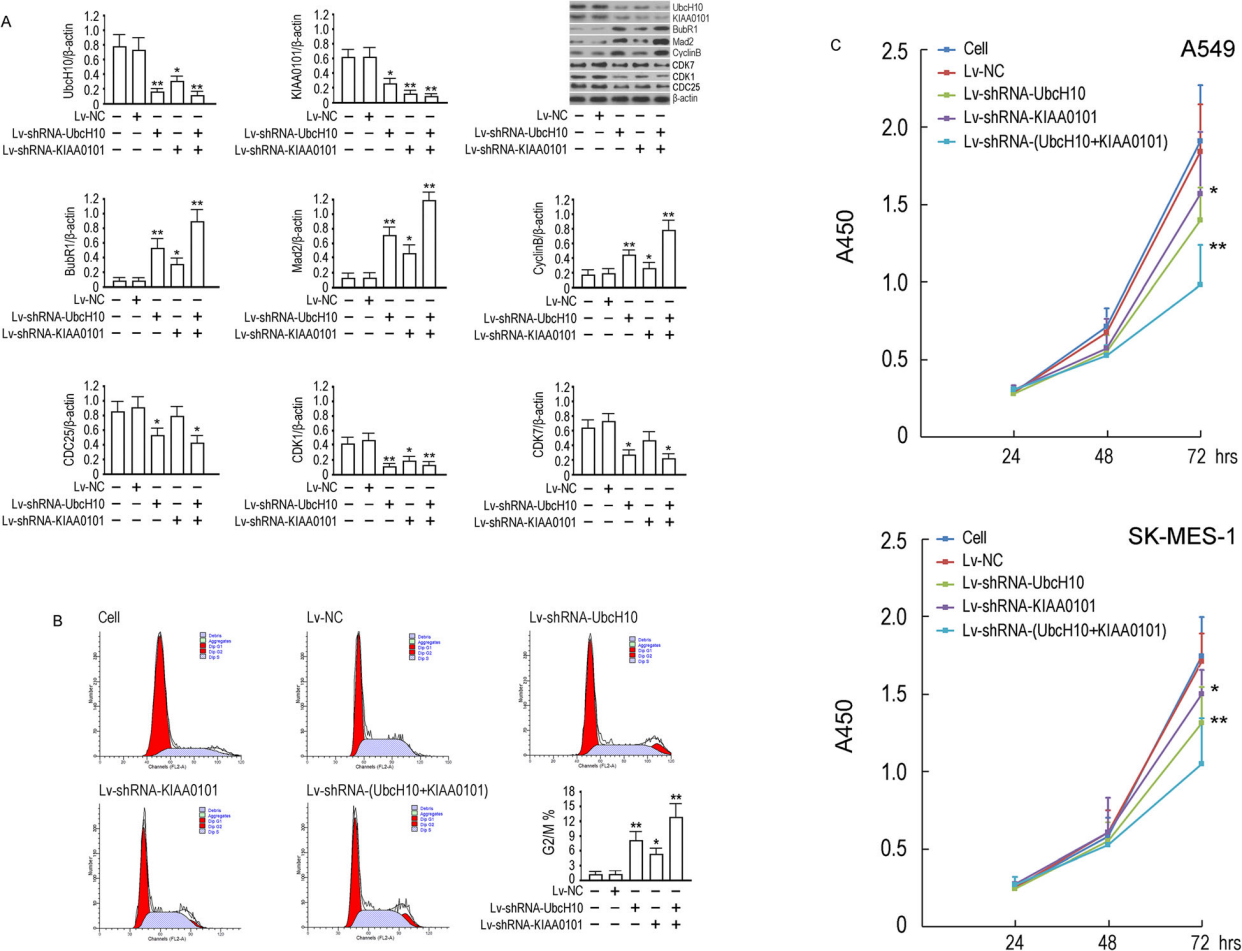
Effect of UbcH10 and KIAA0101 gene knockdown on NSCLC cell SAC components, cell cycle and proliferation. **a** 72 h after lentivirus infection, UbcH10 and KIAA0101 and SAC components BubR1, Mad2 CyclinB, CDC25(72 kDa), CDK1(34 kDa) and CDK7(38 kDa) proteins were detected in SK-MES-1 cells. Scanned target protein bands are shown and the y-coordinate of bar chart represents the relative radio optical density values of proteins to β-actin. **b** Cell cycle analysis of SK-MES-1 cells 72 h after lentivirus infection. **c** The proliferation of A549 and SK-MES-1 cells 72 h after lentivirus infection were determined by CCK-8 assay. The x-coordinate represents the cell grouping and the y-coordinate represents the absorbance at 450 nm which has a strict linear relationship with cell viability. Three biological replicates of all experiments were performed, and the data are expressed as the mean ± SD, **p < 0.01 and *p < 0.05 vs cell group

We first investigated whether UbcH10 or KIAA0101 knockdown influences the SAC and cell cycle-related protein expression in SK-MES-1 cells. The data showed that the suppression of either UbcH10 or KIAA0101 could downregulate BubR1, Mad2 and CyclinB, and upregulate CDC25, CDK1 and CDK7 proteins(*p*<0.05 compared to the control group), and KIAA0101 synergized with UbcH10 to affect the expression of these proteins, with a statistically significant difference between the dual and individual knockdown groups (*p*<0.01 compared to the control group) (Fig. 4a). We then investigated the effects of UbcH10 and KIAA0101 knockdown on the cell cycle. Six hours after the nocodazole treatment was stopped, the G2/M phase ratio was increased significantly among cells with UbcH10 or KIAA0101 knockdown (*p*<0.05 compared to the control group), and the G2/M phase ratio was significantly higher in the co-silenced group (*p*<0.01 compared to the control group) (Fig. 4b). Identical experimental data were obtained in A549 cells (data not shown). We further evaluated the effects of UbcH10 and KIAA0101 knockdown on proliferation and found that A549 and SK-MES-1 cell proliferation was decreased in the UbcH10 shRNA group (*p*<0.05 compared to the control group at 72 h) and significantly decreased in the UbcH10 and KIAA0101 co-silenced group (*p*<0.01 compared to the control group at 72 h) (Fig. 4c). In summary, in NSCLC cells, UbcH10 and KIAA0101 may affect the expression of SAC-related proteins, regulate the cell cycle and promote tumor cell proliferation. Silencing UbcH10 and KIAA0101 can restore SAC function, thus inhibiting the malignant proliferation of tumor cells.

### KIAA0101 may coordinate with UbcH10 and regulate SAC function in a KEN box-dependent manner

**Fig. 5:**
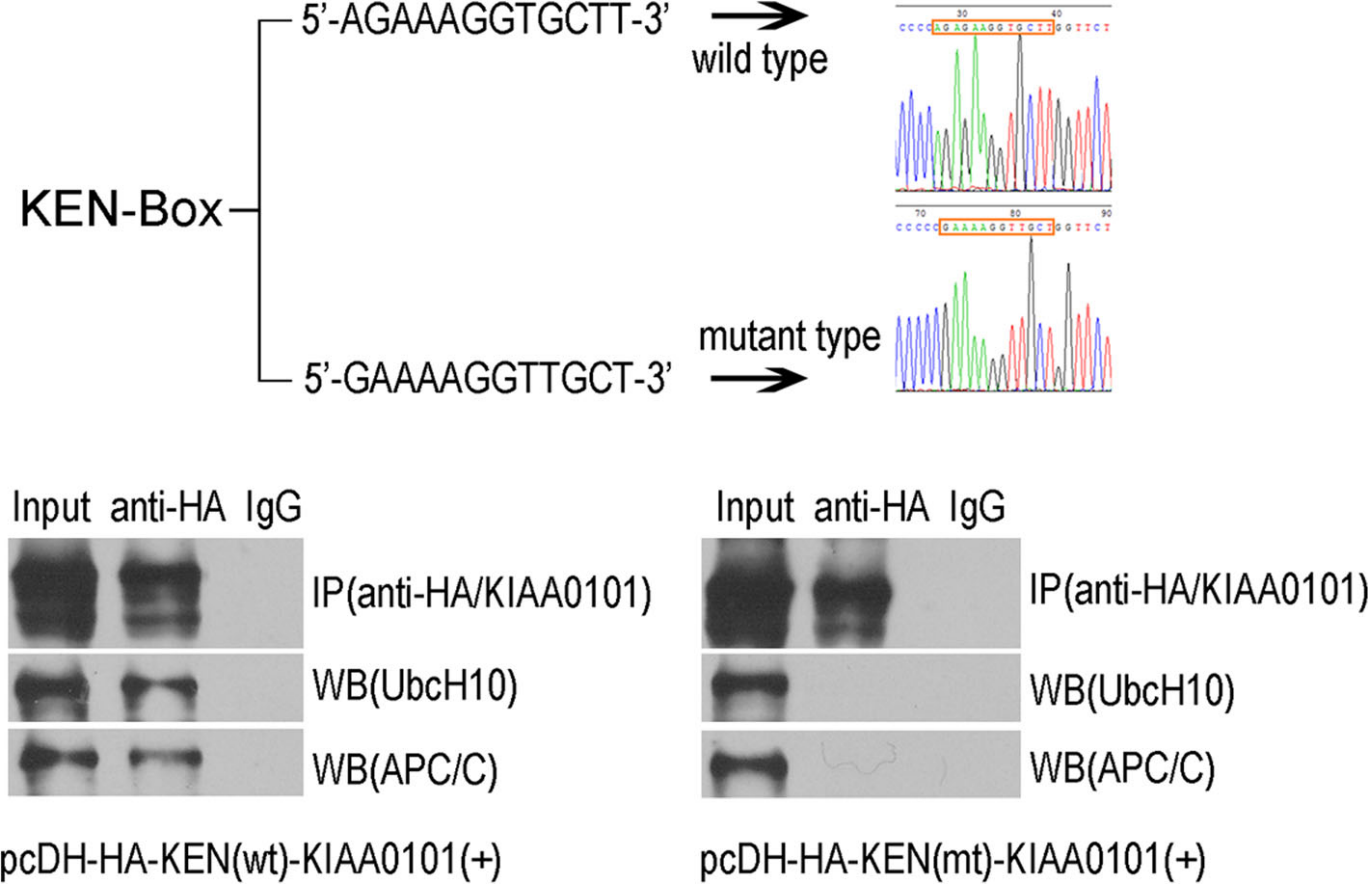
KIAA0101 inhibits the binding of UbcH10 and APC/ C by KEN-box. **a** Construction of the KIAA0101 vector with wild type and mutant KEN-box sites. Left, KEN-box locus and mutation information; right, KEN-box locus sequence analysis; **b** binding of UbcH10 and APC/C was detected by immunoprecipitation. Left, the cells were transfected with the KIAA0101 expression vector containing the wild type KEN-box site, pcDH-KIAA0101 (KEN-wt); right, the cells were transfected with the KIAA0101 expression vector containing the mutant KEN-box site, pcDH-KIAA0101 (KEN-mt). The sample was collected 48 h after transfection. The target APC/C band size was 160 kDa

KIAA0101 and BubR1 share the conserved KEN box sequence that is used to bind the APC/C complex. Therefore, we hypothesized that KIAA0101 may cooperate with UbcH10 to regulate SAC function in a KEN box-dependent manner. Data from coimmunoprecipitation assays showed that KIAA0101 protein containing the wt KEN box bound to UbcH10 and APC/C, while KIAA0101 harboring a mutant KEN box lacked such capability (Fig. 5a, b). Therefore, the ability of overexpressed KIAA0101 in collaboration with UbcH10 to regulate SAC function may be related to KIAA0101 binding to UbcH10 and APC/C via the KEN box.

### Knockdown of UbcH10 and KIAA0101 suppressed the growth of NSCLC in vivo

**Fig. 6:**
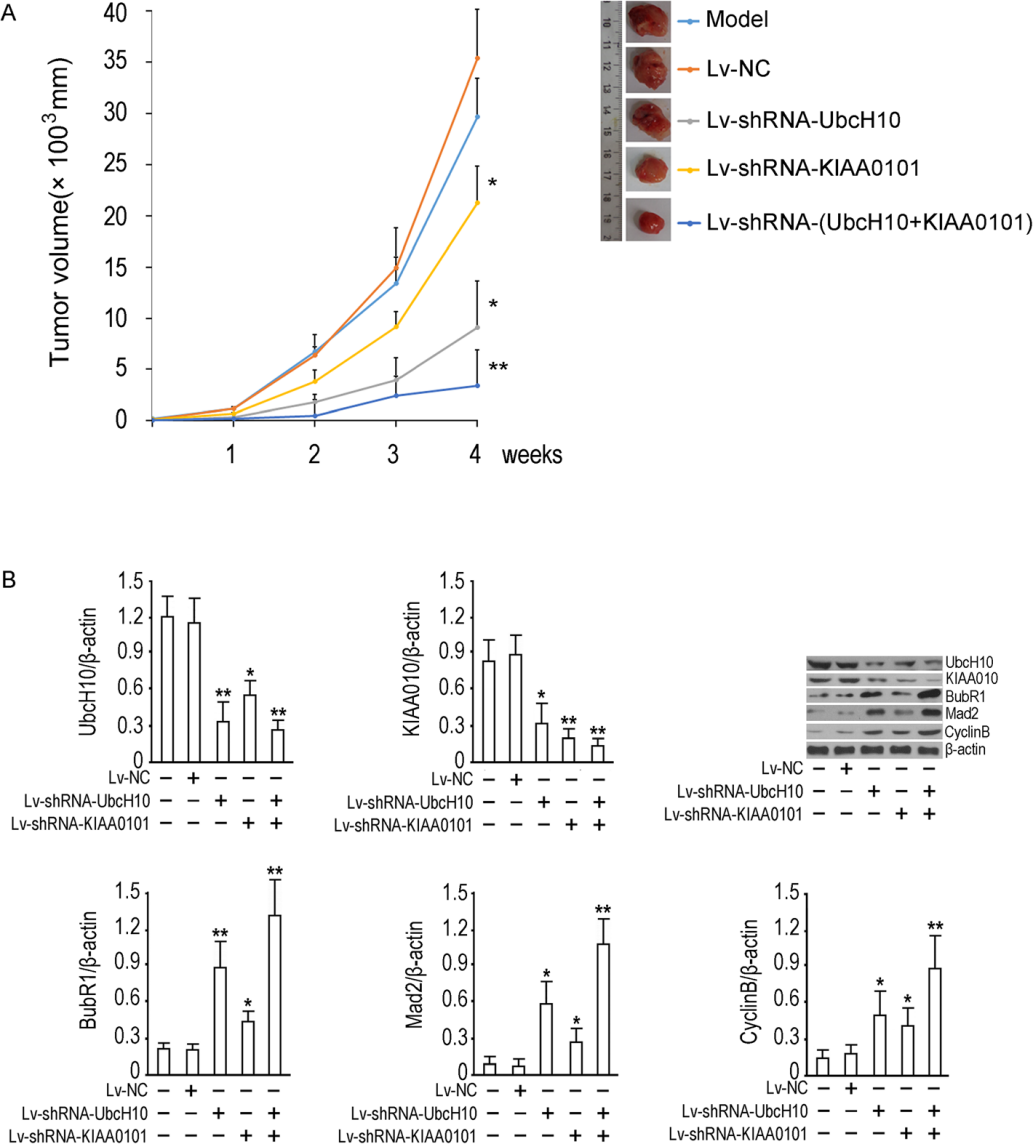
Tumor volume curve and SAC-related functional proteins detection. **a** Tumor volume proliferation rate curve. The tumor volume was calculated as V = 0.5 × a × b × b, with a and b representing the long and short diameter (mm) of the tumor, respectively. The average diameter of the tumors was determined based on the long and short measurements, which were taken 5 times. The parallel group size was set to 12 (*n* = 12). The abscissa is the time after lentivirus injection, and the ordinate is the volume of the tumor. The data are expressed as the mean ± SD. *p < 0.05 and **P < 0.01, vs model group; **b** Western blotting for SAC-related functional proteins. Scanned target protein bands are shown. The y-coordinate of bar chart represents the relative radio optical density values of proteins to β-actin. The data are expressed as the mean ± SD. *p < 0.05 and **p < 0.01, vs model group

We performed further experiments to provide theoretical evidence for UbcH10 and KIAA0101 as therapeutic targets for NSCLC, and the results showed that 4 weeks of intervention had a significant effect on the tumorigenic activity of subcutaneously inoculated NSCLC cells. By week 4, the tumor volume was significantly reduced in the UbcH10 and KIAA0101 shRNA groups (*p*<0.05 compared to the model group), and the co-silenced group showed a superior effect on tumor growth inhibition (*p*<0.01 compared to the model group) (Fig. 6a). We then quantified the expression of related proteins in tumor tissues and found that upon knockdown of UbcH10 and KIAA0101, the expression of the SAC components and cell cycle-associated proteins BubR1, Mad2 and CyclinB was effectively restored in tumor tissues (*p*<0.05 compared to the control group), and the co-silenced group showed a superior effect on these proteins (*p*<0.01 compared to the model group) (Fig. 6b). The results indicated that UbcH10 silencing can restore SAC function to suppress tumor growth in a subcutaneously inoculated NSCLC model. KIAA0101 may play a synergistic role in this process.

## Discussion

Owing to the rapid progression of NSCLC and its high rate of resistance to existing therapies, there remains an urgent need for more effective therapeutic options. In this regard, targeting SAC function has emerged as an attractive therapeutic avenue in the exploration of new therapies for NSCLC. At present, the SAC can be targeted with antitumor therapy in two distinct ways, either by blocking tumor cells from exiting mitosis, which would prolong the mitosis period and eventually lead to cell death, or by promoting premature division during mitosis, thus leading to the formation a numerous aneuploid cells and cell death as a consequence of chromosomal instability [18]. Riccardo Colombo and colleagues found that by selectively inhibiting Mps1, a crucial SAC component primarily responsible for chromosome alignment and the kinetochore–microtubule interaction, effectively inhibited tumor growth in various preclinical cancer models [19]. More recently, Zheng et al. developed a novel Mps1 inhibitor, CFI-402257, and achieved remarkable antineoplastic effects in lung cancer [20]. In the present study, we demonstrated that by targeting UbcH10 and KIAA0101, SAC function could be effectively preserved, and tumor growth could be significantly inhibited, suggesting that UbcH10 and KIAA0101 are potential therapeutic targets in NSCLC that modulate SAC function.

**Fig. 7:**
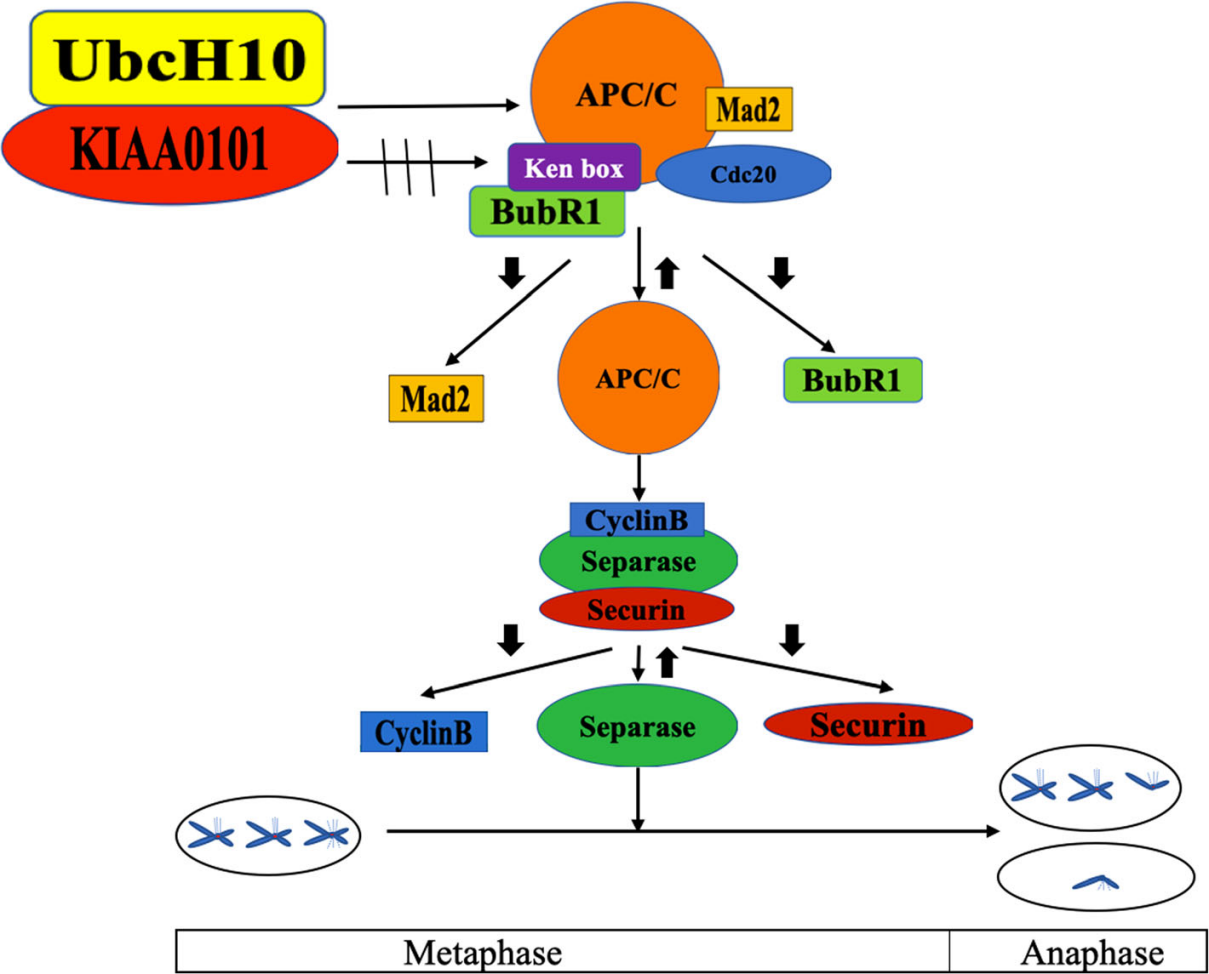
Diagram of KIAA0101 and UbcH110 synergy disrupting the function of the spindle assembly checkpoint in NSCLC cells

UbcH10 is a member of the ubiquitin-conjugating enzyme family and plays a crucial role in the ubiquitin-proteasome pathway (UPP) in humans [14]. During metaphase, proper attachment of the kinetochore to spindle microtubules quenches the inhibitory signals, enabling UbcH10 to mediate the activation of APC/C, which catalyzes the polyubiquitination and destruction of securin and cyclin B. The removal of these mitotic regulators then results in the activation of separase, a clan D protease of the caspase family that initiates anaphase by opening the cohesin ring structures that hold sister chromatids [9]. Reddy S.K. et al. found that in HeLa cells, UbcH10 caused the premature dissociation of Mad2 and BubR1 from the APC/C complex, and free Mad2 and BubR1 molecules could then be recognized by APC/C and degraded by the proteasome [21]. Van and colleagues reported that UbcH10 overexpression leads to global chromosomal instability and the formation of tumors, including lung adenomas [13]. In the present study, we found that UbcH10 affects the expression of the SAC components BubR1 and Mad2 and the cell cycle-related protein CyclinB, resulting in SAC dysfunction and the malignant proliferation of NSCLC cells (Fig. 7). These findings could be partly explained by the fact that in NSCLC, UbcH10 overexpression causes the SAC components Mad2 and BubR1 to prematurely dissociate from the APC/C complex and to be degraded by activated APC/C, which also degrades CyclinB, resulting in mitotic slippage, chromosomal instability and malignant proliferation.

We also discovered that KIAA0101 and UbcH10 expression was spatially and temporally correlated and that KIAA0101 synergized with UbcH10 in regulating SAC function and tumorigenesis. In general, correlated gene expression is mainly due to direct or indirect regulation of one gene product by another gene product, usually a transcription factor. However, there is no direct evidence to indicate that UbcH10 or KIAA0101 can affect the expression of each other as a transcription factor. Therefore, we hypothesized that the temporal and spatial correlation is achieved, to some degree, by regulation of the expression of the KIAA0101 transcription factor by the UbcH10-dependent UPP.

APC/C substrates generally have short peptide motifs, including the destruction box (D box) and the KEN box, that mediate binding to APC/C and are ubiquitinated by APC/C [22]. The SAC members BubR1 and Mad2 could inhibit APC/C activity as pseudosubstrates by occupying substrate binding sites in a KEN and D box-dependent manner. BubR1 and Mad2 dissociate from APC/C and are subsequently degraded, which leads to SAC dysfunction and cell cycle checkpoint termination, eventually causing abnormal chromosomal separation and premature exit from mitosis [23]. KIAA0101 possesses the same sequences for the D box and KEN box sequences as BubR1, which can bind to the APC/C complex [17]. Therefore, we believe that KIAA0101 overexpression may cause the premature dissociation and degradation of BubR1 and Mad2 in part by competing for APC/C binding sites, which in turn leads to SAC dysfunction and malignant proliferation.

UbcH10 gene silencing and UbcH10 and KIAA0101 co-silencing effectively inhibited tumor growth in vivo, and the tumor suppressive effect of co-silencing was slightly better than that of UbcH10 alone, although the difference was not significant. This finding is consistent with our in vitro data. We attribute this outcome to the highly efficient tumor suppression by UbcH10 silencing. Although the difference was not significant, considering the correlated gene expression levels and potential benefits of combined silencing, we believe that UbcH10 and KIAA0101 should be targeted simultaneously for the treatment of NSCLC in the future.

There are still some limitations of our study; most notable, the underlying mechanism of the correlation between UbcH10 and KIAA0101 remains to be elucidated. Based on the results we have already got, we supposed that KIAA0101 induced premature dissociation and degradation of BubR1 and Mad by competing for APC/C binding sites in KEN-box dependent manner, which in turn leads to SAC dysfunction and malignant proliferation. This is similar with mechanisms that UbcH10 modulating SAC function, but this hypothesis requires further validation. Meanwhile, we also found KIAA0101 and UbcH10 expressions were spatially and temporally correlated. This is another crucial mechanism that may explain the interaction between UbcH10 and KIAA0101 though we could not fully elucidate nowadays. Due to the limitations imposed by the experimental conditions, we chose to use a subcutaneous tumor model for the in vivo experiments; however, the results from a spontaneous tumor-forming model with orthotopic cell injection would have undoubtedly been more convincing. Therefore, we intend to use gene editing techniques to knock out the UbcH10 and KIAA0101 genes in a spontaneous tumor-forming model to further support our conclusions.

## Conclusion

In NSCLC, KIAA0101 and UbcH10 interact to cause SAC dysfunction, chromosomal instability and malignant proliferation. KIAA0101 and UbcH10 knockdown reduced the proliferation of NSCLC cells in vitro and suppressed tumor growth in vivo, suggesting that UbcH10 and KIAA0101 are potential therapeutic targets for the treatment of NSCLC by ameliorating SAC function.

## Supplementary information

**Additional file 1.**

**Additional file 2.**

**Additional file 3.**

**Additional file 4.**

**Additional file 5.**

**Additional file 6.**

**Additional file 7.**

## Data Availability

The datasets used and/or analyzed during the current study are available from the corresponding author on reasonable request.

## Abbreviations

SAC: Spindle assembly checkpoint
NSCLC: Non-small cell lung cancer
PBS: Phosphate-buffered saline
GFP: Green fluorescent protein
RT-qPCR: Real-time quantitative PCR
UPP: Ubiquitin-proteasome pathway
