# Supplementary figures and images for: KIAA0101 and UbcH10 interact to regulate non-small cell lung cancer cell proliferation by disrupting the function of the spindle assembly checkpoint
Source: BMC Cancer. 2020 Oct 2;20:957. doi: 10.1186/s12885-020-07463-3 (PMC7532574; doi:10.1186/s12885-020-07463-3)

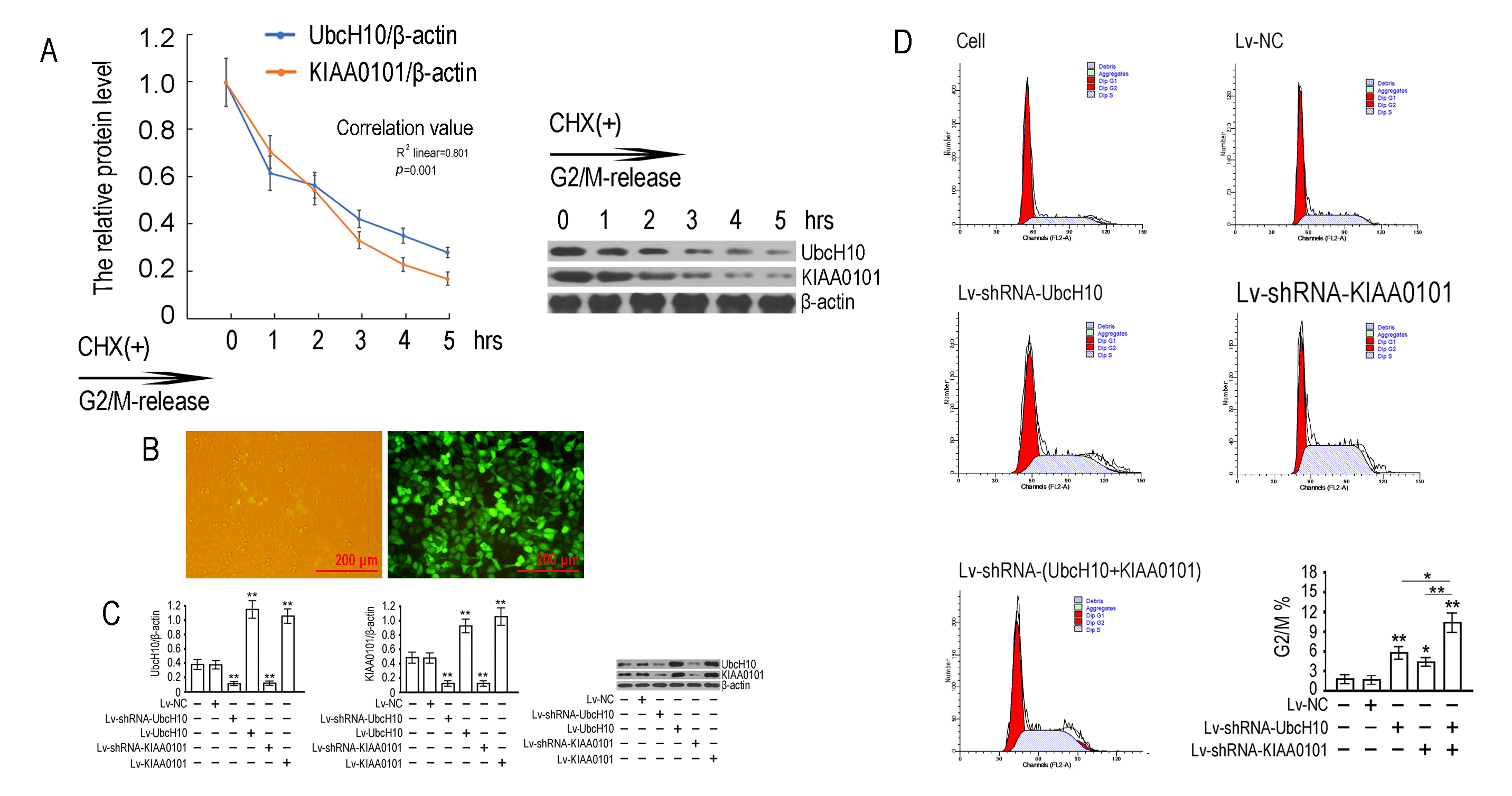

Supplement: Supplementary file 1 — Additional file 1. [file 12885_2020_7463_MOESM1_ESM.tif]

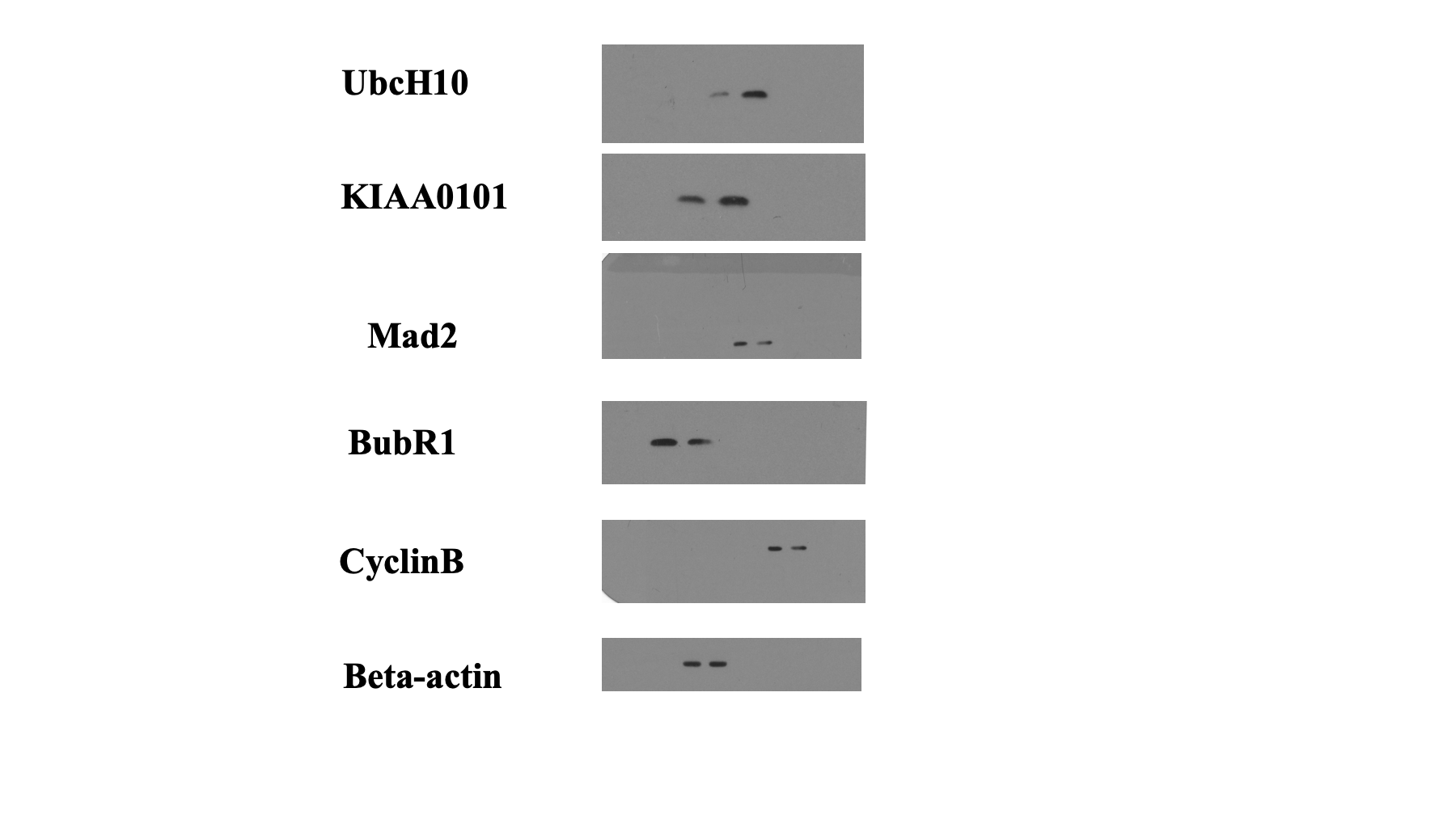

Supplement: Supplementary file 2 — Additional file 2. [file 12885_2020_7463_MOESM2_ESM.tiff]

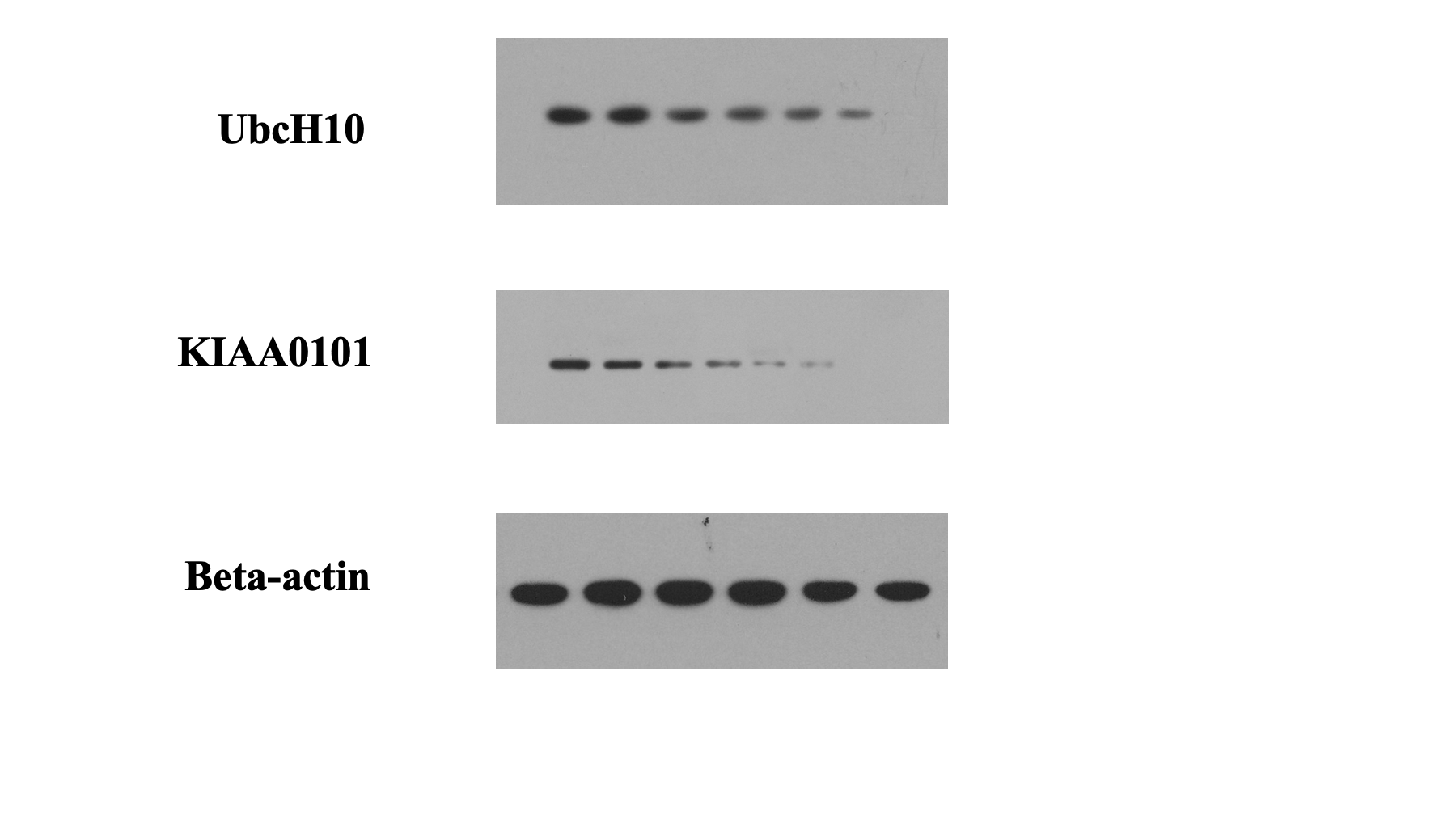

Supplement: Supplementary file 3 — Additional file 3. [file 12885_2020_7463_MOESM3_ESM.tiff]

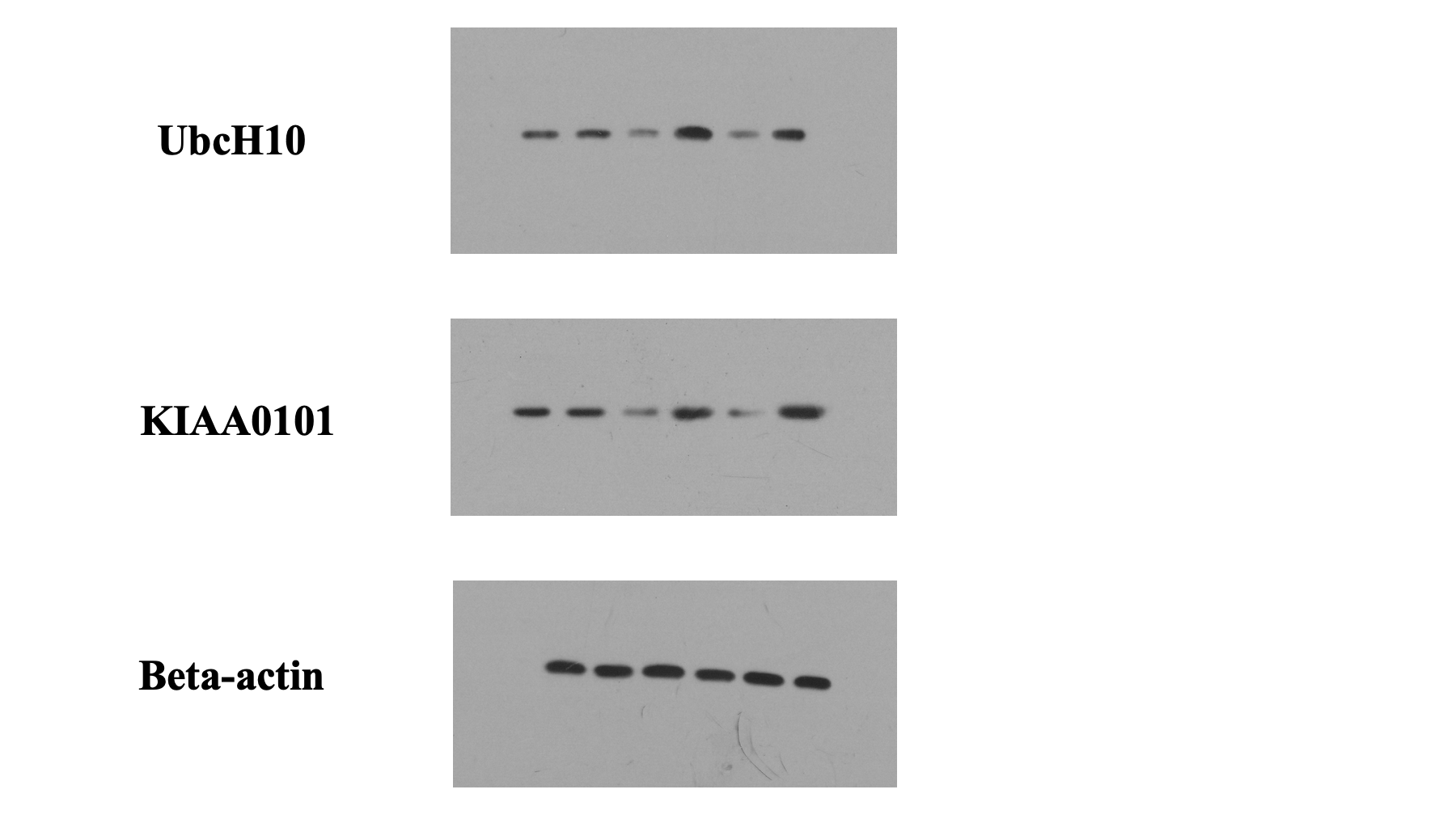

Supplement: Supplementary file 4 — Additional file 4. [file 12885_2020_7463_MOESM4_ESM.tiff]

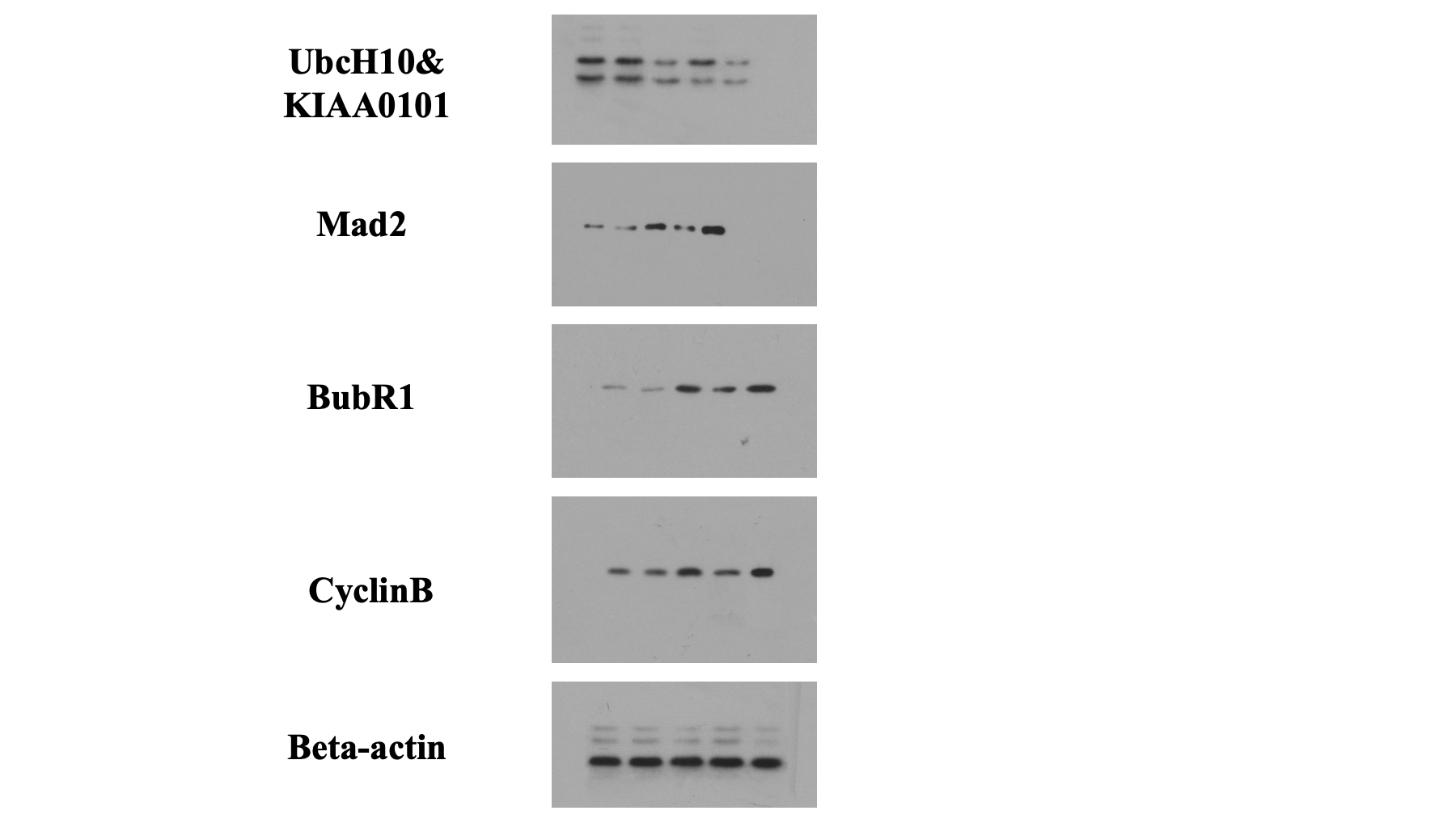

Supplement: Supplementary file 5 — Additional file 5. [file 12885_2020_7463_MOESM5_ESM.tiff]

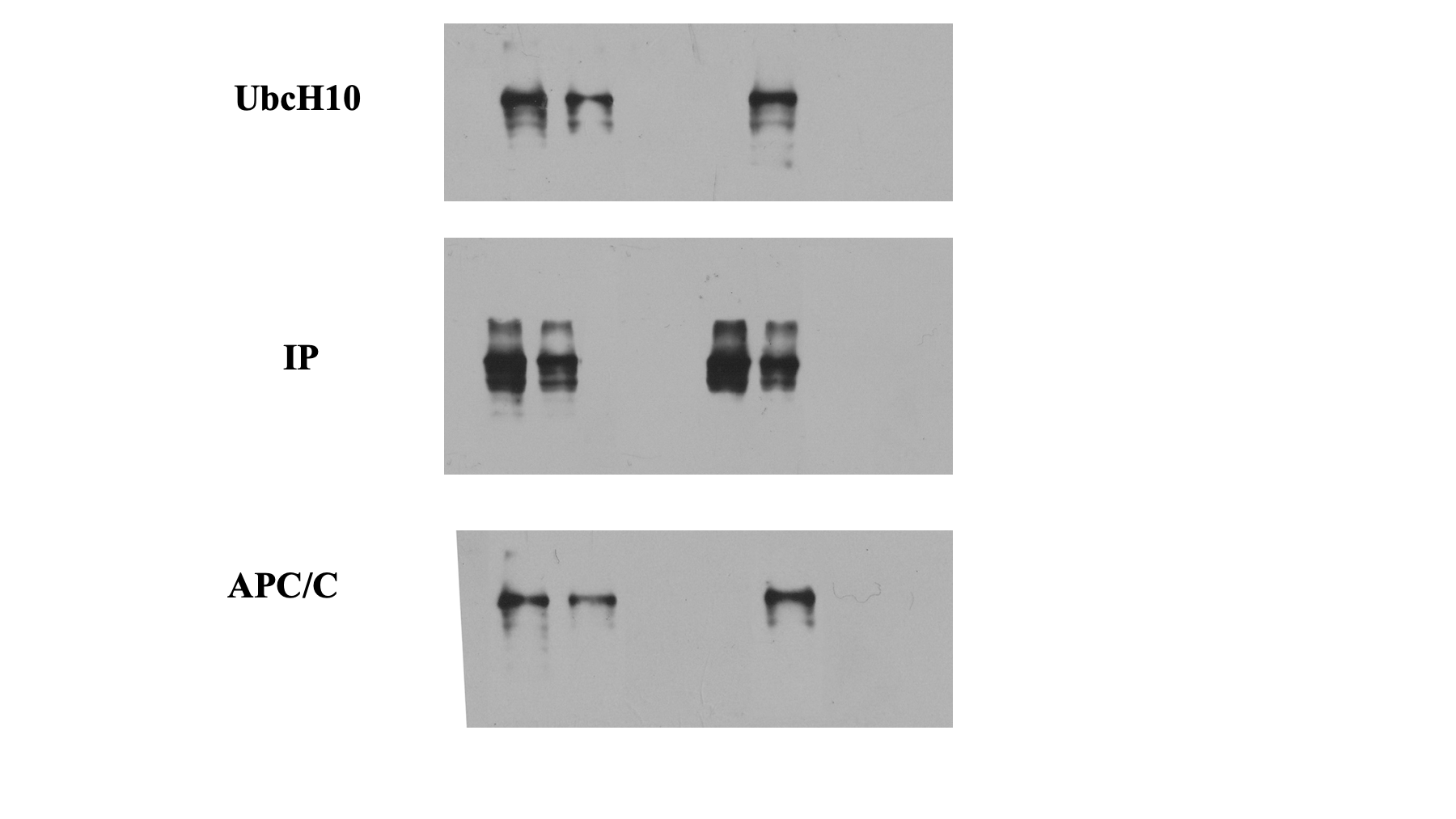

Supplement: Supplementary file 6 — Additional file 6. [file 12885_2020_7463_MOESM6_ESM.tiff]

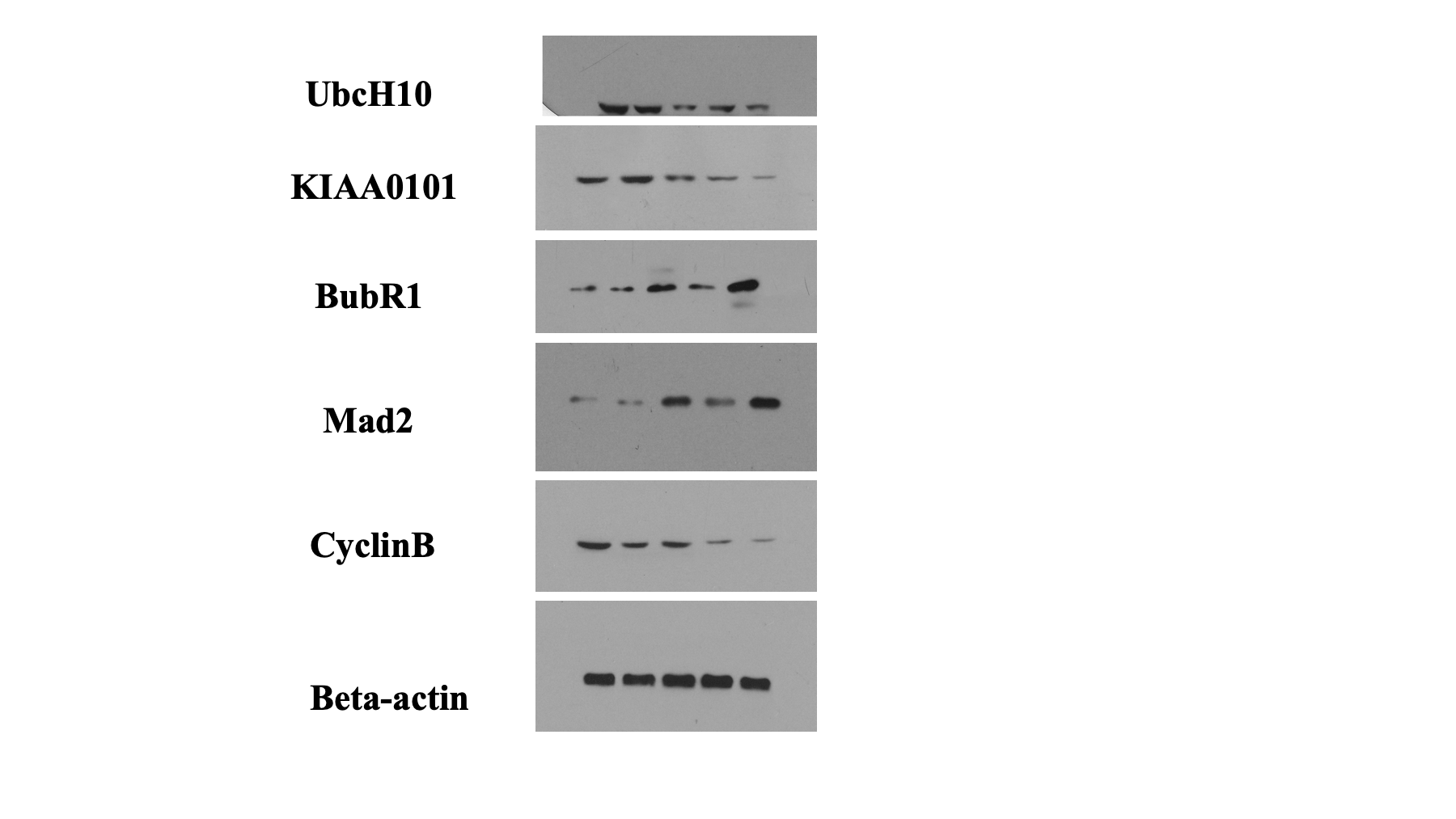

Supplement: Supplementary file 7 — Additional file 7. [file 12885_2020_7463_MOESM7_ESM.tiff]
